# Vocal Accuracy and Neural Plasticity Following Micromelody-Discrimination Training

**DOI:** 10.1371/journal.pone.0011181

**Published:** 2010-06-17

**Authors:** Jean Mary Zarate, Karine Delhommeau, Sean Wood, Robert J. Zatorre

**Affiliations:** 1 Cognitive Neuroscience Unit, Montréal Neurological Institute, McGill University, Montréal, Québec, Canada; 2 International Laboratory for Brain, Music, and Sound Research (BRAMS), Montréal, Québec, Canada; 3 Department of Computer Science, Université de Montréal, Montréal, Québec, Canada; University of Groningen, Netherlands

## Abstract

**Background:**

Recent behavioral studies report correlational evidence to suggest that non-musicians with good pitch discrimination sing more accurately than those with poorer auditory skills. However, other studies have reported a dissociation between perceptual and vocal production skills. In order to elucidate the relationship between auditory discrimination skills and vocal accuracy, we administered an auditory-discrimination training paradigm to a group of non-musicians to determine whether training-enhanced auditory discrimination would specifically result in improved vocal accuracy.

**Methodology/Principal Findings:**

We utilized micromelodies (i.e., melodies with seven different interval scales, each smaller than a semitone) as the main stimuli for auditory discrimination training and testing, and we used single-note and melodic singing tasks to assess vocal accuracy in two groups of non-musicians (experimental and control). To determine if any training-induced improvements in vocal accuracy would be accompanied by related modulations in cortical activity during singing, the experimental group of non-musicians also performed the singing tasks while undergoing functional magnetic resonance imaging (fMRI). Following training, the experimental group exhibited significant enhancements in micromelody discrimination compared to controls. However, we did not observe a correlated improvement in vocal accuracy during single-note or melodic singing, nor did we detect any training-induced changes in activity within brain regions associated with singing.

**Conclusions/Significance:**

Given the observations from our auditory training regimen, we therefore conclude that perceptual discrimination training alone is not sufficient to improve vocal accuracy in non-musicians, supporting the suggested dissociation between auditory perception and vocal production.

## Introduction

Accurate singing requires communication between auditory feedback processing and vocal motor control (i.e., audio-vocal integration) to ensure that each note is produced correctly. During auditory feedback processing, unexpected changes in feedback must be detected, and the mismatch between intended and actual output must be relayed to the vocal motor system for vocal output correction. An enhanced ability to perceive small errors in vocal output may result in more accurate vocal output. To support this, previous behavioral studies by Amir and colleagues and Watts et al. have reported that non-musicians with better pitch discrimination sang more accurately than non-musicians with poorer pitch discrimination [1], . These studies reported a significant correlation between pitch discrimination and vocal accuracy, which suggests that vocal accuracy is partly dependent on auditory perceptual skills – better pitch discrimination may lead to better vocal accuracy in non-musicians. However, Amir et al. and Watts and colleagues did not directly investigate a cause-effect relationship between good auditory skills and accurate singing. Based on this purported relationship, it is reasonable to suggest that enhancing pitch discrimination may improve vocal accuracy in non-musicians. Numerous studies have reported that auditory discrimination training resulted in improved pitch discrimination not only at the training frequency, but at other non-trained frequencies as well [3]–[6]. Additionally, auditory discrimination training with pure-tone stimuli has been shown to improve pitch discrimination with both pure tones and harmonic complex tones [7]. Therefore, if auditory discrimination training improves pitch discrimination overall, this may increase the chance of detecting vocal output errors during singing, which may result in improved vocal accuracy in non-musicians.

Notwithstanding the preceding arguments, other recent studies have reported a dissociation between auditory discrimination and vocal accuracy in non-musicians, regardless of whether they were recruited for poor singing ability [8] or classified according to skill level in either perceptual or production tasks [9], [10]. In order to clarify the discrepancies between these studies and determine if there is a cause-effect relationship between enhanced auditory skills and vocal production, we recruited two groups of non-musicians: a control group and a group that received experimentally-controlled auditory training to enhance their auditory discrimination skills. We tested both groups with auditory discrimination and vocal production tasks both at the beginning and at the end of the experiment to determine if improved auditory discrimination specifically resulted in better vocal accuracy in non-musicians.

Previous studies have demonstrated that short-term training often induces experience-dependent changes in neural activity. For instance, when non-musicians were trained to map particular pitches to piano keys and play short melodies, significant increases in auditory, sensorimotor, frontal, insular, and parietal regions were seen after training [11]–[13]. More importantly, non-musicians who received short-term training in pitch discrimination or auditory working-memory tasks displayed both improved performance in these tasks and enhanced auditory cortical activity after training [14], [15]. Therefore, if short-term auditory discrimination training improves behavioral performance and induces cortical plasticity, a corresponding increase in vocal accuracy may be accompanied by training-induced modulations in activity within a functional network recruited during various singing tasks, which includes auditory cortical regions, motor and premotor areas, insula, thalamus, and cerebellum [16]–[18]. Training-enhanced vocal accuracy may also be paralleled by the recruitment of experience-dependent neural substrates of audio-vocal integration, which include the posterior superior temporal sulcus (pSTS), the anterior portion of the rostral cingulate zone (RCZa), and the anterior insula [18], [19]. The pSTS responds particularly to vocal stimuli [20], [21] and may be recruited specifically to monitor auditory feedback and to detect changes in specific auditory features, including vocal pitch [22]–[24]. The RCZa has been implicated in conflict monitoring in various contexts [25], [26] and may be involved in registering conflicts between the intended notes and actual vocal output. Finally, the anterior insula shares reciprocal connections with these two areas [19], [27], [28] and may contribute to audio-vocal integration by incorporating feedback monitoring processes in the pSTS with conflict detection in the RCZa before a vocal adjustment is made. In an earlier study, we observed that auditory cortex, anterior cingulate cortex, and the insula were functionally connected to each other in both non-musicians and experienced singers, and thus may form a functional network for voluntary vocal pitch regulation [18]. Given that this network was engaged only in experienced singers during vocal pitch regulation tasks in our prior studies [18], [19], training may be needed to consolidate and recruit this network for vocal pitch regulation. Thus, we predicted that these experience-dependent substrates would be recruited following training if subjects used their enhanced auditory skills to ensure accurate singing.

To investigate thoroughly any relationship between auditory discrimination and vocal accuracy and its possible effects on corresponding neural activity, we utilized a multi-step battery of training and testing, and each step had a specific hypothesis. 1) We recruited people with no formal musical experience and separated them into two groups: one group was trained with discrimination tasks to enhance their auditory discrimination skills, while the other group served as controls. Since single-tone discrimination improves after training [3]–[6], we hypothesized that auditory training would also enhance micromelody discrimination. For training, we used pure-tone “micromelodies” with intervals less than 100 cents or one semitone, which is the smallest interval in Western music; training with melodies can lead to improvements in higher-order pattern processing and discrimination [29], [30], rather than enhancements focused only on a single tone. 2) Given that previous studies reported that the effects of auditory training generalized to non-trained frequencies [3]–[6], we expected that training-induced improvements in micromelody discrimination at the training frequency (250 Hz) would also be seen in a non-trained frequency (500 Hz). 3) While we used pure-tone micromelodies for auditory training, we assessed perceptual discrimination skills with both pure- and vocal-tone micromelodies. Any improvements in pure-tone discrimination must transfer to complex-tone discrimination (see [7]), especially vocal tones, in order to conclude that improved auditory skills may result in better error detection in auditory feedback and more accurate singing. Thus, we predicted that training would improve discrimination with both pure- and vocal-tone micromelodies. 4) To test both groups for vocal accuracy, we used two singing tasks: one with single notes (“simple singing”), and another with short, novel melodies with 50- or 100-cent intervals between each note. According to Amir et al.'s and Watts et al.'s findings [1], [2], we hypothesized that if training enhanced micromelody discrimination, then the trained subjects would also be more accurate at singing both single notes and melodies. 5) Simple singing and melodic singing tasks were also used with fMRI techniques to probe the functional networks for singing and audio-vocal integration respectively. Rather than using a pitch-shift paradigm as in our previous experiments [18], [19], we used melodic singing as a more natural and complementary way to target regions of audio-vocal integration, since accurate production of melodies requires audio-vocal integration in a similar fashion as voluntarily correcting for pitch-shifted feedback. When asked to correct for pitch-shifted feedback, a person must monitor the auditory feedback to determine the amount of perturbation before precisely adjusting the vocal output to correct fully for the feedback shift. During melodic singing, the auditory feedback of the currently produced note also must be monitored in order to produce the correct interval to the next note. We hypothesized that voluntarily producing the difficult 50-cent intervals within a melody (“mel50”) would tax the cortical regions involved in audio-vocal integration more than producing 100-cent melodies (“mel100”). During melodic singing prior to training, we expected to see increased activity within auditory and prefrontal cortical regions compared to simple singing, since melodic processing has been reported to recruit these areas for tonal working memory processes [31]–[33]. Additionally, the enhancement of activity within auditory regions during melodic singing may be associated with the processing of more salient pitch changes within auditory feedback [19], , compared to processing the auditory feedback of one note. Since melodic singing requires audio-vocal integration, we hypothesized that singing melodies before training would also recruit the dorsal premotor cortex as a basic substrate for audio-vocal integration (see [18]). This region is involved in forming associations between sensory cues and specific motor commands [36], [37], including auditory-motor interactions during musically-related tasks [38]–[40]. After auditory training, if there were significant changes in vocal accuracy, we expected to see training-induced modulations in cortical activity within the functional network for singing [18], [41] and perhaps also recruitment of experience-dependent substrates of audio-vocal integration, namely the pSTS, RCZa, and the anterior insula [18], [19].

Briefly stated, the present experiment was designed to determine if better auditory discrimination skills would result in improved vocal accuracy in non-musicians, as suggested by both Amir et al. and Watts and colleagues [1], [2], and to assess whether better vocal accuracy would be accompanied by training-induced modifications to the functional network for singing or audio-vocal integration. As detailed below, we determined that auditory discrimination skills generally improved after training, but training-induced changes were not observed in either vocal accuracy or neural activity within the functional networks for singing or audio-vocal integration.

## Results

### Behavioral results

#### Micromelody discrimination task

**Figure 1:**
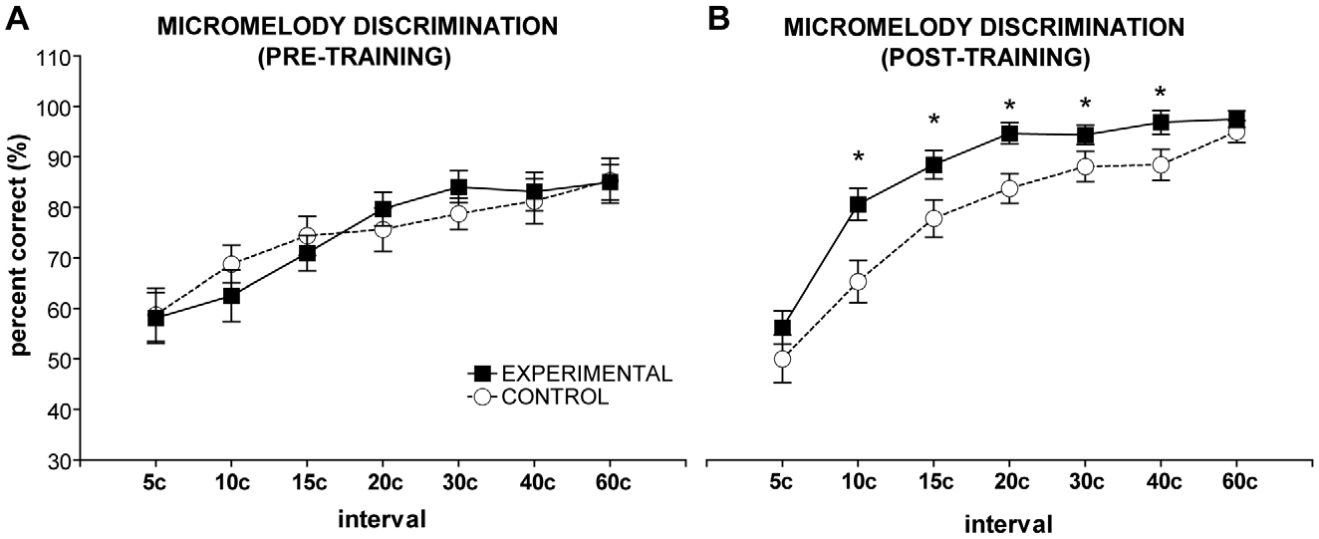
Performance on micromelody discrimination task before and after training. A: Prior to training, both groups of non-musicians performed similarly across all micromelody interval scales. B: After training, the experimental group exhibited better micromelody discrimination with intervals of 10–40 c, compared to controls (denoted by *, all *p*s≤0.05).

The detailed results of the pure-tone micromelody discrimination tasks will be reported in a separate paper (Zatorre, Delhommeau, and Zarate, unpublished work); to summarize, we determined that the experimental group exhibited significantly improved discrimination with pure-tone micromelodies at 250 Hz and other non-trained frequencies compared to controls. Of greatest relevance for the present study are the results from vocal-tone micromelody discrimination, which are not part of the aforementioned paper. The four-way repeated-measures ANOVAs [group (control versus experimental) x frequency (250 Hz versus 500 Hz) x time (pre- versus post-training) x interval scale (seven scales ranging from 5 c to 60 c)] performed on the vocal-tone micromelody discrimination tasks revealed significant main effects of time [F(1,18) = 160.27, *p*<0.001] and scale [F(1,18) = 34.78, *p*<0.001], significant group-by-time [F(1,18) = 46.39, *p*<0.001] and time-by-scale [F(6,108) = 6.47, *p*<0.001] interactions, and a significant three-way interaction between group, time, and scale [F(6,108) = 2.25, *p*<0.05]. No significant main effect of frequency was found (*p*>0.2). Simple effects tests performed on the three-way interaction determined that after a two-week period, the experimental group was better at discriminating vocal-tone micromelodies at 10 c, 15 c, 20 c, 30 c, and 40 c scales than the control group (Fig. 1B; all *p*s≤0.05). Within the experimental group, we found that auditory training significantly improved micromelody discrimination across all scales except the 5 c melodies (all *p*s<0.001). Although the controls did not receive auditory training, they still showed improved discrimination among the 20 c to 60 c micromelodies after two weeks (Fig. 1; all *p*s≤0.01), presumably due to nonspecific factors, but not as much improvement as the trained group displayed at 20 c. The lack of group differences at 5 c and 60 c can be attributed to floor and ceiling effects, respectively. As expected, we also found significant differences in micromelody discrimination across most of the scales within each group at each timepoint (Fig. 1B; all *p*s≤0.01); in general, micromelody discrimination was significantly poorer at the smaller scales (e.g., 5 c to 15 c) than the larger scales (all *p*s<0.05).

#### Simple singing task

The two-way repeated-measures ANOVAs (group by time) performed on the simple singing data found no significant interactions or any significant main effects with respect to vocal accuracy (all *p*s>0.1). Analyses revealed only a marginally significant main effect of time on measures of vocal stability [F(1,17) = 4.43, *p* = 0.05]. During the last behavioral testing session, both groups sang more steadily on single notes, regardless of whether or not they received auditory training (Table 1).

**Table 1:** Measures of vocal stability in simple singing in both groups at each timepoint.

	SESSION 1 Mean ± S.E.	SESSION 2 Mean ± S.E.
control	32.36±5.72	27.21±4.04
trained	28.10±6.06	21.39±2.49
combined	30.34±4.07	24.45±2.46

Both groups sang single notes more steadily during the second testing session at the end of the experiment, compared to the first session (*p* = 0.05).

#### Melodic singing tasks

For a detailed description of each performance measure, refer to the *Materials and Methods*, *Behavioral analyses* section. Table 2 lists all group means for each performance measure taken at each timepoint. After averaging the absolute values of the note errors in each melody (to account for negative and positive errors canceling each other out), the three-way repeated-measures ANOVA [group x time x scale (50 c versus 100 c melodies)] performed on the absolute error measure in mel50 and mel100 tasks revealed only a significant time-by-scale interaction [F(1,17) = 6.85, *p*<0.05]. Simple effects tests found that both groups' mel100 performances were marginally more accurate at the end of the experiment than at the beginning (*p*<0.08), and performance of mel100 tasks was also marginally more accurate than mel50 performance at the end of the experiment (*p*<0.07), regardless of training. No other significant main effects or interactions were observed.

**Table 2:** Melodic performance measures in both groups at each timepoint.

A					
SESS 1	Abs. Error (0)	Abs. Melody Contour Error (0)	Contour Score (4)	Abs. Interval Magn. (50)	Abs. Interval Diff. (0)
control	78.23±13.09	65.99±6.69	3.77±0.06	101.34±6.29	60.15±6.29
trained	65.42±23.55	54.09±9.03	3.67±0.23	92.16±8.27	54.16±13.01
SESS 2					
control	76.67±8.15	69.13±6.25	3.83±0.05	104.89±4.98	61.58±5.51
trained	47.77±8.78	52.90±9.91	3.79±0.18	87.10±4.20	45.28±7.56

B					
SESS 1	Abs. Error (0)	Abs. Melody Contour Error (0)	Contour Score (4)	Abs. Interval Magn. (100)	Abs. Interval Diff. (0)
control	75.36±13.11	47.67±4.24	3.80±0.05	110.42±6.38	42.88±3.54
trained	72.72±27.21	45.91±10.48	3.72±0.20	104.64±8.44	46.64±13.86
SESS 2					
control	68.56±8.72	45.35±4.36	3.86±0.06	113.08±5.10	41.09±4.04
trained	44.52±9.17	39.08±11.07	3.86±0.12	101.28±5.51	35.01±8.26

Group means (± S.E.) are listed for 50-cent (A) and 100-cent (B) melodies, and perfect performance in each measure is indicated in parentheses. For descriptions of how each measure was calculated, refer to the Materials and Methods, Behavioral analyses section.

Following normalization of all produced melodies to determine whether or not subjects produced the correct melody contour, regardless of singing flatter or sharper than the target melody, analyses of the absolute melody contour error showed a significant main effect of scale [F(1,17) = 47.48, *p*<0.001], as well as significant interactions between group and scale [F(1,17) = 4.66, *p*<0.05] and time and scale [F(1,17) = 6.90, *p*<0.05]. Simple effects tests revealed that mel100 tasks were performed more accurately within each group than mel50 tasks (all *p*s<0.01), but there were no group differences in accuracy in each task (all *p*s>0.05). As expected, both groups also performed mel100 tasks more accurately than mel50 tasks at each timepoint (all *p*s<0.001). Additionally, mel100 performance (irrespective of training) improved at the end of the experiment when compared to the first testing session (*p*<0.05), while mel50 performance did not significantly change across both timepoints (*p*>0.1).

Analyses of the contour score (which measures the overall accuracy of upward or downward pitch changes within melodies, regardless of interval size) revealed only a significant main effect of time – contour scores significantly improved in both groups at the end of the experiment [F(1,17) = 4.77, *p*<0.05], regardless of the interval scale. No other significant main effects or interactions were found.

The ANOVA performed on the absolute interval magnitude (50 or 100 cents) resulted in a significant main effect of scale [F(1,17) = 135.22, *p*<0.001] and a significant interaction between group and scale [F(1,17) = 6.18, *p*<0.05]. While there were no significant differences in accuracy between groups for either melody task (all *p*s>0.05), within each group, significantly larger intervals were produced for mel100 tasks when compared to mel50 tasks (all *p*s<0.001), as expected.

After determining the absolute values of produced errors from the target melody interval sizes and directions (i.e., signed intervals: -100 is 100 cents down to next note, +50 is 50 cents up), analyses of the absolute interval difference revealed a significant main effect of scale [F(1,17) = 37.33, *p*<0.001], and significant group-by-scale [F(1,17) = 4.82, *p*<0.05] and time-by-scale interactions [F(1,17) = 4.74, *p*<0.05]. Although group performances did not significantly differ between the two melody tasks, each group performed mel100 more accurately than mel50 (all *p*s<0.05), as expected. Overall, mel100 tasks were performed more accurately than mel50 tasks at each timepoint (all *p*s<0.001), and while mel50 performance did not significantly change across time, mel100 performance slightly improved during the last testing session when compared to the first session (*p*<0.07).

To summarize, we observed significantly enhanced pure- and vocal-tone micromelody discrimination across both frequencies in the group of volunteers who received auditory training, compared to controls. In contrast, we did not detect any significant improvement in vocal accuracy that can be directly attributed to auditory training despite using a variety of vocal performance measures, each of which was sensitive to different aspects of vocal output. Furthermore, all of the regression analyses performed between the discrimination and singing performance measures at each timepoint, as well the regression and correlation analyses performed on the difference scores for each measure between timepoints, did not reveal a significant linear relationship or correlation between micromelody discrimination and vocal accuracy. Therefore, we cannot conclude that enhanced auditory skills resulting from short-term discrimination training are sufficient to improve vocal accuracy in non-musicians.

### fMRI results

#### Functional network for simple singing and effects of training

When comparing imaging data from simple singing trials with data from voice perception trials prior to auditory training, the experimental group recruited a network comprised of auditory cortical regions, sensorimotor cortex, premotor areas, insulae, thalamus, basal ganglia, and cerebellum (Table S1), which is similar to the functional network that we and others previously reported [16]–[18]. Following auditory training, a statistical comparison of the imaging data associated with simple singing from both timepoints revealed no significant training-induced modulations in neural activity.

**Figure 2:**
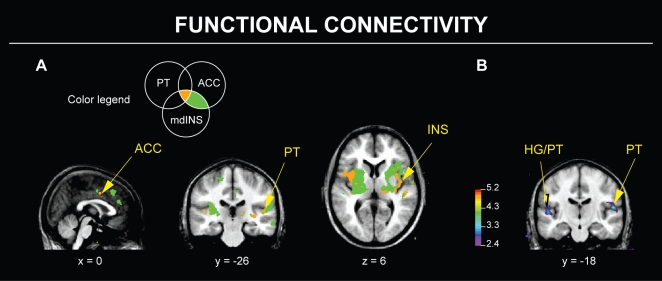
Functional connectivity during simple singing before and after training. A: The different overlap patterns between three connectivity maps during simple singing, generated with seed voxels in right planum temporale (MNI/ICBM152 world coordinates 52, −20, 6), right mid-dorsal insula (40, 4, 6), and left ACC BA 24 (−2, 4, 44); all voxel *p*s≤0.001, uncorrected. The Venn diagram above depicts the color legend used to show overlap in connectivity maps. B: After training, the right planum had stronger connectivity with an adjacent region of right planum and the cortex of the left Heschl's sulcus, which contains Heschl's gyrus and/or planum, during simple singing. All peak/cluster *p*s≤0.05, corrected. ACC  =  anterior cingulate cortex; BA  =  Brodmann area; HG  =  Heschl's gyrus; INS  =  insula; mdINS  =  mid-dorsal insula; PT  =  planum temporale.

To further investigate any training-induced modulations within the functional network for simple singing, we also conducted functional connectivity analyses with seed voxels in the right planum temporale, right mid-dorsal insula, and left anterior cingulate cortex (ACC), Brodmann area (BA) 24, since these regions were significantly active during simple and melodic singing tasks and were also similar to areas that were significantly active during simple singing in a prior experiment [18]. Table 3 and Table S2 show that most of the regions recruited during simple singing are also functionally connected with each other. Furthermore, Table 3, Table S2, and Fig. 2A demonstrate that the connectivity maps associated with each seed voxel (all maps thresholded at *t* = 3.17, uncorrected *p* = 0.001) overlap in the right planum temporale, cortex within the left Heschl's gyrus and sulcus, bilateral insulae, and anterior cingulate cortex, as well as the pars opercularis (BA 44), other frontal areas, and right thalamus. In general, these connectivity maps resemble the connectivity results we outlined in our previous experiments [18], [19]. After comparing functional connectivity maps obtained both before and after auditory training, we observed that the right planum seed voxel exhibited significantly increased functional connectivity with an adjacent region within the right planum temporale, as well as a marginally significant increase in connectivity with the cortex within left Heschl's sulcus after training (Table 3; Fig. 2B).

**Table 3:** Connectivity associated with simple singing.

		A				B			
		*x*	*y*	*z*	*t*	*x*	*y*	*z*	*t*
Auditory	R PAC	50	−14	4	4.6				
	L Planum temporale	−50	−26	12	4.9				
	R Planum temporale	52	−34	14	5.2	54	−20	14	5.2
	L Heschl's sulcus					−52	−16	6	4.8
Motor	L M1	−62	−4	18	4.2				
	L vPMC	−54	4	8	5.3				
Multimodal	L Mid-dorsal insula	−44	−2	10	5.6				
	R Mid-dorsal insula	36	0	2	4.5				
	L Posterior insula	−40	−8	8	5.3				
	R Posterior insula	40	−8	6	4.7				
	L BA 6/44	−50	8	16	4.8				
Frontal	L Inferior frontal - BA 44	−50	10	6	4.7				
	L Frontal operculum	−44	12	4	4.0				
Parietal	R Supramarginal gyrus	62	−32	28	3.6				
Subcortical	R Thalamus	12	−16	16	5.2				
	L Putamen	−32	−8	2	3.9				
	R Putamen	34	−6	4	4.3				

A: Brain regions whose activity significantly correlated to activity within right planum temporale (52, −20, 6) prior to micromelody discrimination training.

B: Brain regions displaying stronger connectivity with the right planum temporale (during simple singing) after auditory training compared to before training. All peak/cluster *p*s≤0.05, corrected. BA  =  Brodmann area; BA 6/44  =  ventral premotor cortex and inferior frontal gyrus, pars opercularis; M1  =  primary motor cortex; PAC  =  primary auditory cortex; vPMC  =  ventral premotor cortex.

#### Functional network for melodic singing and effects of training

Since we did not observe significant differences in neural activity obtained during mel50 and mel100 tasks (results not shown), we subsequently combined the neural activity for both melodic tasks for all other statistical contrasts. Before auditory training, when melodic singing (both mel50 and mel100) was compared with voice perception, subjects engaged a network similar to that observed during simple singing: bilateral auditory cortical regions, sensorimotor cortex, premotor areas, insulae, thalamus, basal ganglia, and cerebellum (Table 4A). When melodic singing was compared with simple singing to isolate regions that were specifically recruited for singing 50 c and 100 c melodies, we found that bilateral auditory regions were significantly more active during melodic singing than singing one note (Table 4B). We also observed increased activity within visual cortical regions during melodic singing compared to simple singing; however, VOI analyses revealed that this was due to a suppression of neural activity within these regions during simple singing, whereas no net change of neural activity occurred in these areas during melodic singing (results not shown). After auditory discrimination training, our statistical comparison of the melodic singing network at both timepoints did not reveal any modulations in neural activity, which is similar to the lack of training-induced plasticity within the network for simple singing.

**Table 4:** Functional network for melodic singing.

		A								B							
		*x*	*y*	*z*	*t*	*x*	*y*	*z*	*t*	*x*	*y*	*z*	*t*	*x*	*y*	*z*	*t*
Auditory	lateral HG	−56	−8	6	9.2					−56	−16	6	6.1				
	aSTG									−56	−2	0	4.6	56	2	−4	3.7
	STG	−54	4	−2	6.6	60	−8	6	8.7	−62	−14	6	6.2	66	−10	2	6.6
	Planum temporale	−56	−24	12	10.2	50	−28	10	8.0	−52	−26	12	6.0	60	−18	4	5.1
Motor	ACC - BA 24	−2	4	44	5.6	4	4	46	5.4								
	ACC - BA 32	−4	10	40	6.2												
	SMA					2	−2	56	8.0								
	M1	−56	−6	26	5.9	52	−8	46	4.3								
	M1 (mouth region)	−46	−14	40	7.4												
	Mid-PMC	−54	−2	46	5.5												
	vPMC	−56	4	26	5.5												
Multimodal	Anterior insula					32	16	12	3.8								
	Mid-dorsal insula	−36	6	8	5.8	40	4	6	3.9								
	Sensorimotor (mouth)	−44	−16	38	7.5	44	−16	40	5.7								
Parietal	Parietal operculum									−42	−34	24	3.6				
Visual	Striate cortex									−6	−80	10	5.0	10	−74	14	6.1
	Lingual gyrus									−10	−60	0	3.7				
	Inferior occipital													36	−76	−16	3.9
Subcortical	Thalamus	−12	−24	−2	5.0	18	−14	6	4.9								
	Putamen	−28	−4	−4	4.8	22	−6	8	5.1								
	Lat. globus pallidus					22	−12	6	5.1								
Cerebellum	Declive (VI)	−16	−62	−20	6.2	10	−62	−20	5.0								
	Vermis (V/culmen)					2	−56	−18	3.7								
	Vermis (VI/declive)					2	−60	−24	4.1					32	−70	−22	4.4

A: Regions of peak neural activity (divided by hemisphere: -x values represent regions in the left hemisphere) during both melodic singing tasks compared with voice perception, prior to training.

B: Regions of peak neural activity (divided by hemisphere) during both melodic singing tasks compared with simple singing, prior to training. All peak/cluster *p*s≤0.05, corrected. ACC  =  anterior cingulate cortex; aSTG  =  anterior superior temporal gyrus; BA  =  Brodmann area; HG  =  Heschl's gyrus; M1  =  primary motor cortex; mid-PMC  =  mid-premotor cortex; SMA  =  supplementary motor area; STG  =  superior temporal gyrus; vPMC  =  ventral premotor cortex.

To determine whether melodic singing differentially enhanced connectivity within the functional network for singing compared to simple singing, we conducted analyses of stimulus-modulated functional connectivity (with the same seed voxels listed above) on the imaging data acquired before auditory training. We did not find any significant enhancements of connectivity specifically attributed to melodic singing, so we therefore conclude that similar patterns of functional connectivity are recruited during simple singing and melodic singing. Following training, our volunteers did not exhibit any training-dependent enhancements in connectivity specifically due to melodic singing (compared with simple singing); hence, we further conclude that micromelody discrimination training did not affect neural activity during melodic singing.

## Discussion

### Behavioral results

As predicted, the group that received auditory training with pure-tone micromelodies exhibited significantly enhanced discrimination with both pure-tone and vocal-tone micromelodies at the training frequency (250 Hz) and a non-trained frequency (500 Hz), compared to controls (see Fig. 1). We evaluated vocal accuracy with a simple pitch-matching task (i.e., simple singing) and more thoroughly with an array of melodic singing performance measures that ranged from assessing global accuracy via the contour score to much finer evaluation with the absolute error from each target melody; however, we did not observe any significant differences between the trained group and controls at the end of the experiment. We only found a marginally significant improvement in vocal stability during simple singing in the final testing session, as well as more accurate performances of the 100 c melodies compared to 50 c melodies, but because there were no significant group differences in these findings, we attribute this to nonspecific effects and/or task familiarization. Finally, we did not find a significant linear relationship or correlation between micromelody discrimination skills and vocal performance. Accordingly, we cannot conclude that training-enhanced auditory discrimination specifically results in improved vocal accuracy in non-musicians.

The lack of experimental evidence to support Amir et al.'s and Watts and colleagues' proposed relationship between enhanced auditory skills and good vocal accuracy resembles the dissociation between auditory perceptual and vocal production skills reported by Bradshaw and McHenry, Dalla Bella and colleagues, and Pfordresher and Brown [8]–[10]. Each of these studies observed poor vocal accuracy in non-musicians who possessed good auditory discrimination skills. In addition, despite their reported correlation between perception and production, Watts and his team still found that some non-musicians who sang inaccurately possessed comparable auditory discrimination skills to non-musicians who sang accurately [2]. While these observations may be attributed solely to imprecise vocal control accompanied by good auditory skills, Amir and colleagues reported that a few non-musicians with poor discrimination skills sang accurately [1]. Therefore, the purported correlation in these two studies between auditory discrimination and vocal accuracy cannot fully account for all variability in singing ability. Furthermore, since the studies cited above have reported non-musicians with poor vocal accuracy and good auditory discrimination, as well as non-musicians who sing accurately with poor discrimination skills, the observed dissociation between perception and production cannot be explained solely by deficient auditory skills or imprecise vocal control. Rather, the dissociation may be due to a sensorimotor deficit, in which good auditory discrimination and vocal production skills may exist, but a correctly perceived auditory target or model is incorrectly coupled with a vocal motor command [10].

It should be noted that the aforementioned studies attempted to define a relationship between perception and production (or a lack thereof) simply based on evaluations of discrimination skills and vocal accuracy. In contrast, we directly intervened with performance in one of these domains by using a discrimination training paradigm to specifically enhance auditory discrimination skills, and then we assessed the training effects on both perceptual and production skills. The result of this direct manipulation, which has not previously been attempted to our knowledge, strengthens our conclusion of a dissociation between auditory discrimination ability and vocal accuracy in non-musicians.

### fMRI results

#### Basic functional network for singing prior to micromelody discrimination training

Broadly speaking, the functional network recruited during simple singing (compared to voice perception) obtained before discrimination training replicated the results that we found during simple singing tasks in our previous studies [18], [19] and other singing tasks [16], [17]. Furthermore, the overlapping connectivity patterns obtained during simple singing resembled the patterns of connectivity observed in both of our previous studies, even though different methods, singing tasks, and subject groups with varying singing expertise were used to generate these connectivity maps. Together, the replication of functional connectivity results across all three studies suggests that the temporal neocortex (within the STG, pSTS, or planum temporale), anterior cingulate cortex, insula, and other regions within the functional network for singing can interact with each other during various single-note singing tasks.

Prior to micromelody discrimination training, we determined that the regions engaged during simple singing are also recruited during melodic singing, when compared to voice perception. As predicted, when we compared the imaging data acquired during both types of singing tasks, we discovered that melodic singing recruited more activity within bilateral auditory cortex than simple singing. A previous neuroimaging study by Brown and colleagues also determined that melodic singing required more auditory cortical activity than monotonic vocalization, during which the same note was sung repeatedly to control for the same number of notes in both tasks [42]. We attribute this increase in auditory cortical activity to the salience of pitch changes within auditory feedback during melodic singing [19], [34], [35], as opposed to the feedback of one note during simple singing. Notably, neither our study nor Brown et al.'s study observed enhanced prefrontal or parietal cortical activity due to tonal working memory processes, as suggested by several prior studies [32], [43], [44]. However, in those studies, subjects performed specific pitch-comparison tasks requiring them to hold one pitch in memory while ignoring interfering sounds, which may impose a greater working memory load than our melodic reproduction task; thus, the lack of a large cognitive load and/or the significant task differences between our study and previous research may account for the absence of prefrontal or parietal activity during our melodic singing task. Since melodic singing did not enhance connectivity within the functional network for singing before auditory training (relative to simple singing), we also conclude that this functional network is involved in both single-note and melodic singing tasks to a comparable degree.

#### Evaluating the functional network for singing following micromelody discrimination training

When we compared the imaging data from before and after training, we did not observe any significant training-induced modulations in activity within the functional network for singing during any singing task. Subsequently, we conducted connectivity analyses as a potentially more sensitive method to detect training-induced plasticity within the network. We determined that following training, the right planum temporale had stronger intra- and interhemispheric functional connectivity with auditory cortical regions (i.e., right planum temporale and cortex within the left Heschl's sulcus). The significance of this finding remains to be established, but it may be related to a marginally significant improvement during simple singing – at the end of the experiment, subjects exhibited less pitch variability during this task. However, this behavioral change cannot be specifically attributed to discrimination training, since both the experimental and control groups showed this improvement. We may only speculate that repeated exposure to the simple singing task, regardless of training, may improve vocal performance over time, which may also be accompanied by enhanced connectivity between auditory regions.

Since micromelodies were used as the main stimulus for discrimination training, we expected to see the strongest training effects on neural activity measured during melodic singing. However, we did not observe any training-induced neural plasticity during melodic singing. The lack of training-induced neural changes may be explained by the concurrent absence of improvements in vocal accuracy during melodic singing. In contrast, not only did training significantly improve micromelody discrimination across different frequencies compared to controls, but this improvement in behavior was also accompanied by training-induced modulation in auditory cortical activity and right prefrontal regions (Zatorre, Delhommeau, and Zarate, unpublished work). Hence, there is a clear correspondence between training-induced neural changes and the related behavior: when behavior improves after training, there is also a concomitant modification of neural activity, whereas in the present study there was no change in either behavior or brain activity.

It may be argued that the absence of training-induced improvements in vocal accuracy and related modulations in neural activity may be attributed to a lack of statistical power, sensitivity in our experimental design, or insufficient length of training. However, we can address these concerns in a few ways. First, the number of subjects in each group provided enough statistical power to detect significant improvements in micromelody discrimination in the experimental group compared to controls, as well as corresponding training-induced modulations in auditory cortical activity (Zatorre, Delhommeau, and Zarate, unpublished work). Second, these training-related changes were observed after only six sessions, demonstrating that our training paradigm was sufficient to significantly affect micromelody discrimination and related neural activity. Third, our vocal performance measures were sensitive enough to reveal a marginally significant improvement in vocal stability in the last testing session, even though this improvement was seen in both groups. Finally, our inclusion of a control group was absolutely essential; without this group, we would have incorrectly attributed the improved vocal stability at the end of the experiment as a training effect. Therefore, we can be assured that our observations suggest that vocal accuracy is not affected by training-enhanced auditory discrimination, which implies a dissociation between auditory perceptual and vocal production skills in non-musicians. However, we cannot conclude that auditory perception and vocal production are fully independent of one another, since previous experiments have demonstrated that perturbations in auditory feedback can result in an immediate vocal correction, such as the pitch-shift response [45]–[48] or the Lombard effect [49], [50]. Additionally, the lack of auditory training-induced improvements in vocal production may be attributed to different timecourses of training-enhanced performance in each domain. Following auditory training, improvements in vocal accuracy may not have manifested until some time after our last behavioral testing session. Future experiments may need additional testing sessions after auditory training to determine whether vocal accuracy improves at a slower rate than auditory discrimination. Alternatively, vocal accuracy may only be improved following auditory and vocal training, during which non-musicians can be trained to map target pitches correctly to a vocal motor sequence. Indeed, following musical training, both instrumental and vocal musicians exhibited better auditory discrimination and vocal accuracy, and instrumentalists in particular demonstrated a significant positive correlation between these skills [51]. In light of this, our experimental results provide evidence that auditory perception and vocal production are only partly dissociable in non-musicians.

### Conclusion

The present experiment was designed to determine whether enhancements in auditory discrimination after training would result in improved singing accuracy and corresponding neural plasticity within the functional network for singing. We determined that non-musicians who received training exhibited better micromelody discrimination skills than controls. Nevertheless, we did not find corresponding improvements in vocal accuracy or modulations in singing-related cortical activity following training. We therefore conclude that training-enhanced auditory skills are not sufficient to improve vocal accuracy in non-musicians.

We observed that auditory cortex, anterior cingulate cortex, and insula are functionally connected during singing tasks. In general, this resembles the patterns of functional connectivity seen both in non-musicians and experienced singers in our previous singing studies [18], [19]. In our recent paper [19], we suggested that these regions may be increasingly recruited for audio-vocal integration as a function of vocal training and practice. Although we found increased functional connectivity among auditory cortical regions during simple singing after auditory training, we did not see corresponding increases in connectivity among the other regions of this hypothesized network for audio-vocal integration. This lends further support to our notion that short-term auditory training alone is not sufficient to improve vocal accuracy or recruit a potential network of audio-vocal integration that may assist in improving vocal accuracy. Based on Pfordresher and Brown's sensorimotor-deficit model of poor singing [10], we suggest that audio-vocal training may be necessary to ensure that auditory targets are matched with the correct vocal motor commands to reproduce the presented notes or melodies. We propose that only after joint auditory and vocal motor training would vocal accuracy improve, and that this improvement would then be accompanied by training-related plasticity within the functional network for audio-vocal integration.

## Materials and Methods

### Ethics Statement

All subjects gave written informed consent to participate in this study, in accordance with procedures approved by the Research Ethics Committees of the McConnell Brain Imaging Centre and the Montréal Neurological Institute.

### Subjects

A total of 20 healthy subjects were recruited from the McGill University community. All subjects (mean age  = 22±4.4 years old) were right-handed, had normal hearing, and were devoid of neurological or psychological disorders and contraindications for functional magnetic resonance imaging (fMRI) techniques. All subjects were classified as non-musicians because they had less than three years of vocal and/or musical training or experience and were not currently practicing or performing music. The 20 subjects (12 female) were randomly divided into two groups of 10 people each – an experimental group that received auditory training and one control group that did not receive any training.

### Order of tasks

**Figure 3:**
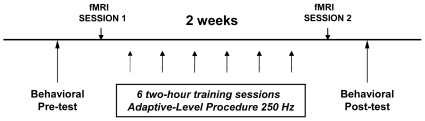
General timeline for all testing and training sessions. At the beginning of the experiment, all subjects were tested with singing and auditory discrimination tasks. A few days later, the experimental group (designated for auditory training) performed a subset of these tasks again in the MR scanner. Following the scanning session, the experimental group received six sessions of auditory discrimination training spread across two weeks. The experimental subjects were scanned again, performing the same subset of tasks as in the first scanning session. Finally, both the experimental and control groups were tested again with auditory discrimination and singing tasks.

Fig. 3 depicts the general timeline for all testing and training sessions. The training group was tested with vocal production tasks and then auditory discrimination tasks to determine baseline performance levels. Then, these subjects performed a subset of these auditory and vocal tasks in the magnetic resonance scanner to obtain neuroimaging data. After the first scanning session, the experimental group was given auditory discrimination training across six sessions spread over two weeks (for training details, see *Auditory Discrimination Training and Tasks*). Following training, these subjects once again performed a subset of auditory and vocal tasks in the scanner. Finally, the trained subjects were behaviorally tested one last time, first with auditory discrimination tasks and then vocal production tasks. Control subjects were tested only with the more extensive battery of auditory and vocal tasks twice, in the same task order as the trained subjects, with approximately 16 days between the sessions to match the amount of time between initial and final behavioral testing in the trained subjects.

### Equipment

For the behavioral testing sessions, each subject was seated comfortably in front of a lab computer screen and given a pair of lab headphones (Sony MDR-V900, New York, NY, USA), through which all auditory stimuli were delivered. During the auditory discrimination tasks, all stimuli were presented using Presentation software (Neurobehavioral Systems, Inc., Albany, CA, USA). For the vocal production tasks, subjects were given a lab microphone (Audio-Technica ATM41HE, Stow, OH, USA), which was connected to a mixer to amplify the voice signal before it was sent to a VoiceOne digital signal processor (TC-Helicon Vocal Technologies, Westlake Village, CA, USA). The digital signal processor turned off auditory feedback in the headphones at the end of each trial to simulate auditory settings during scanning sessions, in which auditory feedback was cut off before image acquisition to prevent scanner noise from entering the headphones. The vocal tasks were controlled using Media Control Functions (MCF) software (DigiVox, Montréal, QC, Canada). Auditory feedback (via the digital signal processor) and all vocalizations were digitally recorded onto a Marantz PMD-670 digital recorder (D&M Professional, Itasca, IL, USA). During scanning sessions, subjects in the training group were tested in a Siemens Sonata 1.5-Tesla magnetic resonance (MR) scanner. Each subject was given MR-compatible headphones with an attached MR-compatible microphone (Commander XG headset, Resonance Technology, Inc., Northridge, CA, USA) before lying down on the scanner bed. All visual cues were back-projected onto a screen at the subjects' feet, viewed via a mirror attached to the head coil.

### Stimuli

**Figure 4:**
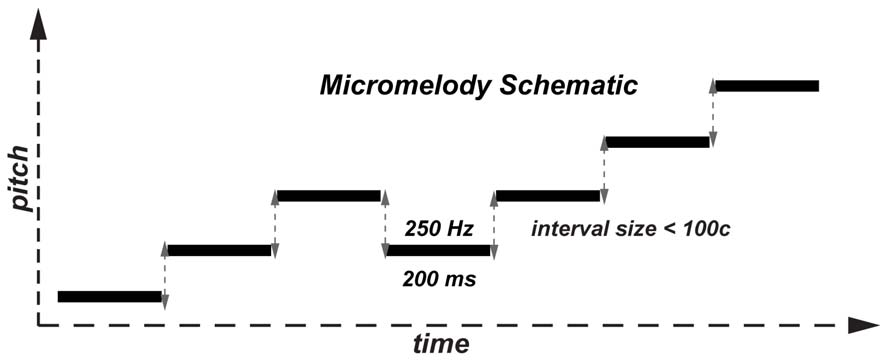
Schematic of the micromelodies used for auditory discrimination training and testing. Each micromelody consists of seven notes (each 200 ms in length; ISI of 150 ms between each note not illustrated), with the pitch of the middle note always at 250 Hz (the training frequency). The intervals between notes (in double-headed, dashed arrows) were always less than 100 cents (or a semitone).

We used micromelodies as the main stimuli for auditory discrimination training and testing. We define micromelodies as seven-note melodies with intervals (frequency ratios) that are smaller than 100 cents (a semitone in musical terminology, which is the smallest interval in the Western musical scale). Each micromelody was 2.35 seconds in length – each note was 200 ms long, with an internote interval of 150 ms, and 50 ms of silence at the end of the melody; there was an interstimulus interval of one second within each pair of micromelodies presented for discrimination. The micromelodies were constructed via MATLAB with either pure tones or vocal tones (recordings of a male or female voice singing /a/), and the middle note of each micromelody was either at the training frequency of 250 Hz or the non-trained frequency of 500 Hz (Fig. 4). In general, all micromelodies were constructed so that there would be either two or three inversions of melodic contour [e.g., down-down-down-**up**-**down**-down-**up** would contain three inversions (denoted in boldface)] and either zero or one consecutive repetition of a note (e.g., the third and fourth notes are the same in a zero-repetition melodic contour).

Amir and colleagues suggested that further investigations of auditory perception and vocal production should use the same fundamental frequency to evaluate auditory discrimination and vocal accuracy, and then determine whether any relationship established at that particular frequency also applied to other frequencies [1]. Therefore, since the training frequency was 250 Hz, our target note for all singing tasks was also approximately 250 Hz, which is within the vocal range of both men and women. The presented targets used either a male or a female voice singing the syllable /a/ at 252 Hz (or ∼B3) or melodies with the middle vocal tone always at 252 Hz.

During all singing tasks, pink noise was delivered through the headphones to reduce bone conduction and MR scanner noise (during scanning sessions). All auditory stimuli (pink noise, target vocal waves, and auditory feedback) were delivered to the headphones via the mixer, and all volume levels were adjusted to comfortable levels for each subject. Pink noise was delivered at an average of 70.8 dB SPL A, while the target waves were presented at an average of 8.9 dB above the pink noise.

### Auditory discrimination tasks and training

Our auditory discrimination task was a two-alternative, forced-choice procedure in which subjects were presented with two micromelodies in succession and were required to indicate whether they were the same or different; during discrimination testing, subjects did not receive feedback on whether or not their answer was correct. We used seven different interval scales for testing: 5- 10-, 15-, 20-, 30-, 40-, and 60-cent micromelodies; micromelodies at each interval scale were randomly presented during testing. On half of the discrimination trials, the micromelodies within a presented pair were the same, and on the other half, the micromelodies were matched for interval scale (e.g., both with 20 c intervals) but had different melodic contours (i.e., more than one note differed between the two micromelodies).

**Figure 5:**
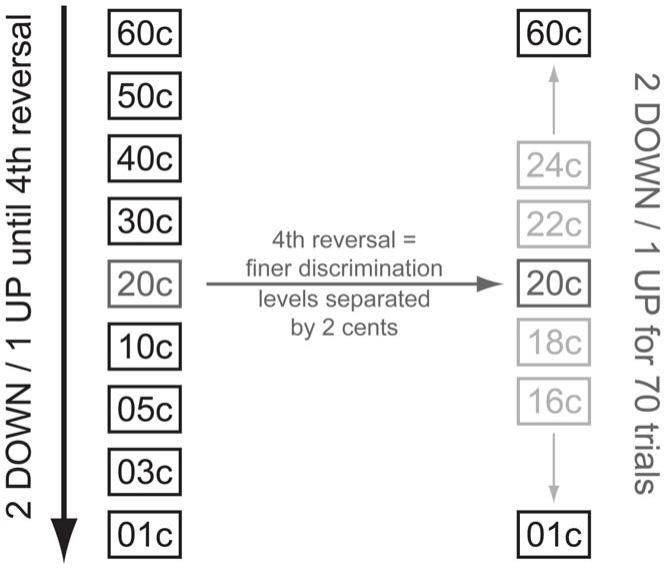
The “2 down – 1 up” adaptive level variation rule used for micromelody discrimination training. For every two correct discrimination trials, the size of the micromelody interval decreased by one level (e.g., 60 c to 50 c), thus making the discrimination task more difficult. If a trial was answered incorrectly, the micromelody interval increased by one level. After four reversals of difficulty level, 70 trials of training for finer discrimination began at the interval scale that elicited the fourth reversal (indicated in dark gray). During this finer discrimination training, the adaptive-level rule was applied again, but each level was separated by only two cents.

After the first behavioral and fMRI testing sessions, the experimental group went through six training sessions of pure-tone micromelody discrimination at 250 Hz, spread across two weeks. As opposed to testing sessions, subjects received feedback on their answers. Additionally, we presented micromelodies with more interval scales ranging from 1 c to 60 c. Training sessions used a two-alternative, forced-choice procedure with a “2 down-1 up” adaptive level variation rule (Fig. 5) – after two successive trials that were correctly answered, the difficulty level would increase (e.g., go down from 60 c to 50 c melodies), and for each trial that was answered incorrectly, the difficulty level would decrease (e.g., go up from 50 c to 60 c melodies). This adaptive-level procedure would continue until four reversals in difficulty occurred, resulting in a variable number of trials per subject in each session. Subsequently, finer discrimination training occurred over 70 trials, starting at the interval size that evoked the fourth reversal; each difficulty level was separated by only two cents during this portion of training.

Following training, subjects were tested again for pure- and vocal-tone micromelody discrimination centered at 250 and 500 Hz to determine whether training with pure-tone micromelodies at 250 Hz would also improve discrimination with micromelodies at 500 Hz and micromelodies with vocal tones at both frequencies. This procedure was identical to the micromelody tasks presented before training.

### Vocal production tasks and fMRI protocol

For all singing tasks, we first presented a target note or melody and then used a visual cue to prompt the subjects to sing the note or melody back. All subjects were trained to sing using the syllable /a/ with minimal mouth movement to reduce movement artifacts in the fMRI session – they were instructed to keep their jaws slightly open and lips closed, so that at the beginning and end of every sung note, only their lips, but not their jaws, moved. Each of three singing tasks was presented in blocks of five trials. In the first task, after hearing the two-second target note, subjects were cued to sing the note for four seconds (“simple singing” condition). The second and third singing tasks involved singing five-note melodies centered on the target frequency (252 Hz). In one melody task, melodic intervals were 50 cents (“mel50”), while the intervals were 100 cents in the other melody task (“mel100”). During behavioral testing sessions, the subjects went through four experimental runs with all tasks included in each run, and all tasks were counterbalanced across subjects. The target melodies used in the melodic singing tasks were pseudo-randomized within each melody-singing block.

A few days after the behavioral testing session, each subject in the experimental group performed all singing tasks in the MR scanner. In addition to these tasks, four control conditions were also presented – a condition with only pink noise playing in the background, used to assess “baseline” cortical activity, and three voice perception tasks that presented either the target note or melodies (at 50 c or 100 c intervals) that subjects did not have to sing back, thus serving as an auditory control for each singing condition in the scanner. In all control conditions, subjects were visually cued to breathe out normally, rather than sing; therefore, these conditions also served as a respiratory control for the singing conditions.

Prior to functional scanning, a high-resolution (voxel  = 1 mm^3^) T1-weighted scan was obtained for anatomical localization. During the two functional runs, one whole-head frame of twenty-five contiguous T2*-weighted images were acquired in an interleaved fashion [TE = 85 ms, TR = 10 s, 64×64 matrix, voxel size  = (5×5×5)mm^3^, FOV = 320 mm^2^]. We utilized a sparse-sampling experimental design, in which tasks were performed during the silences between image acquisitions to prevent scanner noise from interfering with the auditory stimuli and to reduce any effect of movement [52]. Timings of task presentations were systematically varied or “jittered” by +/−500 ms to maximize the likelihood of obtaining the peak of the hemodynamic response for each task. Within each run, each of the singing and control conditions was presented 10 times, with one scan acquisition per condition. Each subject went through two experimental runs in the scanner, resulting in a total of 20 acquisitions per condition. As in the behavioral testing sessions, the order of the conditions within each run was counterbalanced across subjects, and the five target melodies were pseudo-randomized within each melodic condition.

### Behavioral analyses

For both the control and experimental groups, micromelody discrimination performance at each frequency (250 and 500 Hz) was assessed at each timepoint (pre- versus post-training) by determining the percentage of trials that each subject answered correctly. The percentages were analyzed using four-way repeated-measures analyses of variance (ANOVAs), with group as the between-subjects variable and frequency, time, and micromelody interval scale (e.g., 5 c, 10 c, etc.) as within-subject variables. Simple effects tests were used to analyze all significant interactions, and the Bonferroni *t*-test was used for all post-hoc analyses.

For vocal production analyses, we automated the statistics extraction process using Python in conjunction with de Cheveigné's MATLAB implementation of the YIN pitch extractor [53]. For each vocalization, YIN was used to calculate fundamental frequency (*f0*), signal power and aperiodicity every 32 samples [resulting in a frame rate of 1378.125 Hz, i.e., (44.1/32) kHz]. Since YIN normally calculates *f0* in octaves relative to 440 Hz, we modified the code to determine *f0* relative to the target vocal tone of 252 Hz and then multiply each *f0* value by 1200 to convert to cents (one octave equals 1200 cents); this conversion normalized the vocal data for comparison across genders, testing sessions, and subject groups (experimental versus control). Subsequently, the mean *f0* and standard deviation were calculated for each vocalization during simple singing.

**Figure 6:**
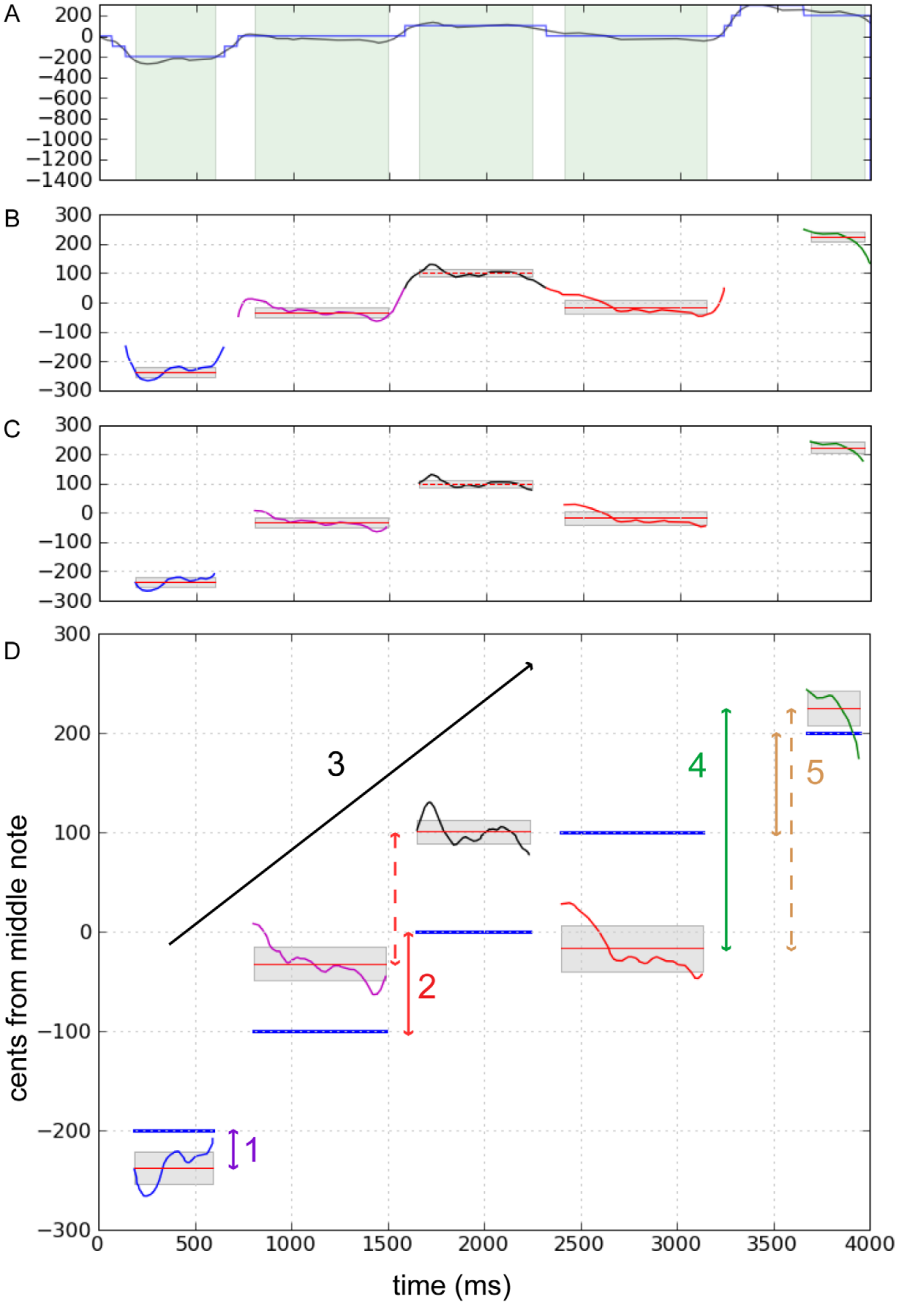
Segmentation and performance measures applied to sung melodies. For all plots, the x-axis represents time in milliseconds, and the y-axis represents vocal pitch converted to cents, relative to the middle note (252 Hz). A: The raw pitch trace (black line) of a non-musician's sung melody, including sliding transitions between notes. The blue line represents time segments at which the Viterbi algorithm detected steady-state vocal pitch; a new segment begins when the algorithm detects a significantly different vocal pitch. B: The algorithm is programmed to take the five longest pitch segments (depicted in five different colors), which usually corresponds to the five sung notes within the melody. To reduce the pitch variability due to note transitions, we calculated the mean (thin red horizontal line) and standard deviation (gray shaded boxes above and below the mean) from the middle 80% of the note, which is represented by the full horizontal extent of the mean/standard deviation [and the green vertical boxes in (A)]. C: This trace shows the complete results of the melody segmentation with five distinct notes. D: This graph shows the non-musician's produced melody (five differently colored segments) and the target melody, represented by blue horizontal lines. The different numbers show the different performances measures we used to evaluate vocal accuracy: 1) average of the absolute values of error from target melody (purple arrows); 2) average of the absolute values of error from the target melody contour (solid red arrows measure target melody intervals, dashed red arrows represent produced melody intervals); 3) contour score, which measures the number of pitch changes correctly produced (in this case, all target melody notes go up; black arrow); 4) absolute interval magnitude that was produced (green arrows); and 5) average of the absolute values of differences from the signed intervals of the target melody (solid tan arrows measure target melody intervals, dashed tan arrows represent produced melody intervals). To distinguish between performance measures (2) and (5), the second measure is derived by subtracting the produced pitch of the middle note from the entire sung melody and the resulting melody contour is then compared to the target melody contour, whereas in measure (5), the direction (i.e., the +/− sign) and magnitude of the produced intervals are compared with those in the target melody.

For melodic singing, we segmented the vocalizations into their constituent notes. This was done using the Viterbi algorithm [54], which is a dynamic programming algorithm that allows us to determine the longest segments of time during which the *f0* is approximately constant. Prior to running the Viterbi algorithm, the *f0* vector (i.e., *f0* across time) was first expanded into a state matrix with one column per sample: each column contained a smoothed version of *f0*, and each row represented a particular frequency. The Viterbi algorithm was then run on the state matrix to determine the most likely path, which was the most likely *f0* state for each column. During time periods with small *f0* changes, the path remained in the same *f0* state (Fig. 6A). Therefore, we defined the notes of each sung melody as the five longest segments during which the state remained unchanged (Fig. 6B). To reduce *f0* variability due to transitions between notes, the middle 80% of each segment was used to calculate mean *f0* and standard deviation (Fig. 6C). Finally, the resulting segmentations were verified by visual inspection. Approximately 10% of segmentations were determined to be incorrect (e.g., segments containing more than one note, one note divided into two segments) and were not included in subsequent analyses.

After initial processing, the resulting vocal data from the three singing tasks were handled slightly differently. Here, we present the vocal data from the two behavioral sessions to determine whether experimentally-controlled auditory training improved vocal accuracy within the trained non-musicians compared to controls. For simple singing, we calculated the average error from the target note (vocal accuracy measured in cents) and average vocal stability (i.e., average of standard deviations across trials in cents – smaller standard deviations reflect greater vocal stability), and used these measures as dependent variables. Each variable was entered into two-way repeated-measures ANOVAs [group by time (first and last testing sessions)]. For melodic singing tasks, we calculated five different performance measures (Fig. 6D): 1) average of the *absolute values* of error from target melody (absolute values taken to correct for negative and positive errors canceling each other out – “absolute error” should be 0); 2) average of the absolute values of error from the target melody contour, after normalizing produced melodies for pitch height (i.e., singing lower or higher than a melody centered at 252 Hz; “absolute melody contour error” should be 0); 3) number of pitch changes out of four that were produced in the correct direction (e.g., up, down, up, down), when compared to target melody (“contour score” out of four); 4) average of the absolute value of the interval produced between notes, regardless of direction (“absolute interval magnitude” should be 50 for 50 c melodies, 100 for 100 c melodies); and 5) average of the absolute values of differences from the signed intervals of the target melody, which measures errors from both interval size and direction, regardless of how flat or sharp the melody was compared to the target (“absolute interval difference” should be 0). Each of these performance measures were analyzed by three-way repeated-measures ANOVAs [group by time by scale (50 c or 100 c)]. Simple effects tests were used to analyze all significant interactions, and the Bonferroni *t*-test was used for all post-hoc analyses.

In order to determine if there was a significant linear relationship between auditory perceptual and vocal production skills, we performed regression analyses between micromelody discrimination performance and either simple singing or melodic performance measures at each timepoint. To assess if training-enhanced micromelody discrimination specifically led to changes in vocal performance, we also calculated difference scores between the two timepoints (beginning and end of the experiment) to assess improvement in each of these perceptual and production measures, and then used these difference scores in regression and correlation analyses.

### fMRI analyses

To correct for motion artifacts, all blood-oxygen-level-dependent (BOLD) images were realigned with the fourth frame of the first functional run using the AFNI software [55]. To increase the signal-to-noise ratio of the imaging data, the images were spatially smoothed with an 8-mm full-width at half-maximum (fwhm) isotropic Gaussian kernel. Prior to analysis, the first four frames were excluded from further analyses to remove T1-saturation effects; these frames were acquired either during practice singing trials or presented instructions. For each subject, we conducted our image analyses in a similar fashion to that described in our first paper [18] using fMRISTAT, which involves a set of four MATLAB functions that utilize the general linear model for analyses [56]. Before group statistical maps for each contrast of interest were generated, in-house software was used to nonlinearly transform anatomical and functional images from each subject into standardized stereotaxic coordinate space, using the nonlinearly-transformed, symmetric MNI/ICBM 152 template [57]–[59]. To determine session effects (i.e., post-training versus pre-training), we first statistically compared the post-training data with the pre-training data using a fixed-effects linear model in each subject. We subsequently combined these results across all subjects with a mixed-effects linear model. Lastly, the program *stat_summary* reported the minimum *p*-values among those computed with a Bonferroni correction, random field theory, and discrete local maxima [60]. We report peaks of neural activity that survived the critical *t*-threshold corrected for multiple comparisons, which *stat_summary* determined to be 5.0 at *p* = 0.05, using a whole-brain search volume. While some peaks did not meet the critical threshold, they fell within regions previously reported in one of our earlier studies [18]. For these *a priori* regions, we corrected the threshold for small volumes and reported peaks if their corrected voxel or cluster *p*-values were equal or less than 0.05.

We performed conjunction analyses between the group-averaged images acquired during simple singing and both melodic singing tasks (compared to voice perception) to choose the proper seed voxels for connectivity analyses. For conjunction analyses, we utilized an in-house tool called *mincmath* to find the minimum *t*-statistic at each voxel across the images for all singing tasks. The conjunction results were then tested against the “conjunction null hypothesis”, which entailed using the critical *t*-values for just one contrast, to determine whether there was significant neural activity in certain brain regions in all singing tasks [61]. We then chose seed voxels in the right planum temporale, right mid-dorsal insula, and the left anterior cingulate cortex (BA 24) since they were significantly recruited during all singing tasks in this experiment. Furthermore, these seed voxels are in similar regions that were also used as seed voxels for functional connectivity analyses in our first experiment [18].

In our analyses of functional connectivity, the general linear model was fitted to account for the neural activity due to a stimulus (e.g., any singing task). Then, the remaining activity within a specific voxel (the “seed” voxel) was regressed on the activity within the rest of the brain (on a voxel-by-voxel basis) to determine where activity significantly covaries with the activity at that seed voxel, without the effect of a stimulus [62], [63]. We also performed analyses of stimulus-modulated functional connectivity, which assessed how the connectivity is affected by the stimulus or task of interest [64]. Using *stat_summary*, the critical *t*-threshold for all connectivity analyses was determined to be 4.8 (*p* = 0.05, corrected for multiple comparisons).

Voxel-of-interest (VOI) analyses were performed on voxels that displayed peak activity in group-contrasted BOLD images. For each voxel in MNI/ICBM space, the BOLD signal is extracted from the same voxel in standardized space within each subject. At each VOI, the BOLD signal for the task-of-interest is calculated as a percentage of change of BOLD signal during the baseline condition in the following way: [(BOLD signal during task – BOLD signal during baseline)/(BOLD signal during baseline)] x 100%.]

The locations of peak neural activity or connectivity were classified using: 1) neuroanatomical atlases [59], [65], [66]; 2) probabilistic maps or profiles for the Heschl's gyrus [67], planum temporale [68], mouth region of the sensorimotor cortex [69], inferior frontal gyrus pars opercularis [70], and basal ganglia [71]; and 3) locations defined by previous reports or reviews on the medial frontal and cingulate areas [72], [73] and subdivisions of the premotor cortex [39].

### Data exclusions

For vocal analyses, 510 out of 5640 vocalizations were excluded due to equipment failure, subject-performance error, or problems with pitch extraction or melody segmentation. For fMRI analyses, 72 out of 1920 frames were excluded from analyses due to equipment failure or performance errors only. One subject from the experimental group did not complete the fMRI sessions and was subsequently excluded from all data analyses.

## Supporting Information

Table S1 Regions of peak neural activity during the simple singing task compared with voice perception, prior to training. All peak/cluster ps≤0.05, corrected. ACC  =  anterior cingulate cortex; BA  =  Brodmann area; M1  =  primary motor cortex; PAC  =  primary auditory cortex; SMA  =  supplementary motor area; STG  =  superior temporal gyrus; vPMC  =  ventral premotor cortex.
(0.07 MB DOC)

Table S2 Connectivity maps associated with right mid-dorsal insula and left ACC BA 24 during simple singing. All peak/cluster ps≤0.05, corrected. ACC  =  anterior cingulate cortex; BA  =  Brodmann area; dPMC  =  dorsal premotor cortex; IPL  =  inferior parietal lobule; L  =  left; M1  =  primary motor cortex; mid-PMC  =  mid-premotor cortex; PAC  =  primary auditory cortex; post  =  posterior; pre-SMA  =  pre-supplementary motor area; pSTG  =  posterior superior temporal gyrus; pSTS  =  posterior superior temporal sulcus; R  =  right; RCZa  =  anterior portion of rostral cingulate zone; SMA  =  supplementary motor area; STG  =  superior temporal gyrus; STS  =  superior temporal sulcus; vPMC  =  ventral premotor cortex.
(0.09 MB DOC)
