# Supplementary material for: Vocal Accuracy and Neural Plasticity Following Micromelody-Discrimination Training
Source: PLoS One. 2010 Jun 17;5(6):e11181. doi: 10.1371/journal.pone.0011181 (PMC2887372; doi:10.1371/journal.pone.0011181)
Supplement: Table S1 — Regions of peak neural activity during the simple singing task compared with voice perception, prior to training. All peak/cluster ps≤0.05, corrected. ACC = anterior cingulate cortex; BA = Brodmann area; M1 = primary motor cortex; PAC = primary auditory cortex; SMA = supplementary motor area; STG = superior temporal gyrus; vPMC = ventral premotor cortex. (0.07 MB DOC) [file pone.0011181.s001.doc]

Table S1. Regions of peak neural activity during the simple singing task compared with voice perception, prior to training.

|  |  | **SIMPLE - PERCEPTION (PRE)** | | | | | | | |
| --- | --- | --- | --- | --- | --- | --- | --- | --- | --- |
|  |  | **LEFT** | | | | **RIGHT** | | | |
|  |  | ***x*** | ***y*** | ***z*** | ***t*** | ***x*** | ***y*** | ***z*** | ***t*** |
| Auditory | PAC | -46 | -20 | 4 | 8.2 | 48 | -22 | 6 | 7.6 |
|  | STG | -54 | -8 | 6 | 8.7 | 58 | -16 | 4 | 8.1 |
|  | Planum temporale | -48 | -26 | 12 | 9.5 | 54 | -28 | 10 | 7.2 |
|  |  |  |  |  |  |  |  |  |  |
| Motor | SMA |  |  |  |  | 2 | -2 | 58 | 7.5 |
|  | ACC - BA 32 | -2 | 8 | 42 | 6.5 | 4 | 16 | 32 | 3.7 |
|  | ACC - BA 24 |  |  |  |  | 2 | -4 | 42 | 3.9 |
|  | M1 |  |  |  |  | 54 | -8 | 46 | 6.2 |
|  | vPMC | -60 | 0 | 28 | 5.3 | 60 | 0 | 34 | 3.4 |
|  |  |  |  |  |  |  |  |  |  |
| Multimodal | Anterior insula | -36 | 14 | 10 | 6.0 |  |  |  |  |
|  | Mid-dorsal insula | -34 | -2 | 8 | 4.7 | 38 | 4 | 6 | 5.8 |
|  | Posterior insula |  |  |  |  | 38 | -10 | -6 | 4.5 |
|  |  |  |  |  |  |  |  |  |  |
| Frontal | Frontal operculum | -34 | 16 | 12 | 5.7 | 34 | 16 | 12 | 4.3 |
|  |  |  |  |  |  |  |  |  |  |
| Parietal | Postcentral (mouth region) | -46 | -14 | 40 | 6.3 | 44 | -16 | 40 | 7.3 |
|  | Subcentral gyrus | -56 | -8 | 18 | 5.9 |  |  |  |  |
|  | Supramarginal gyrus | -58 | -36 | 34 | 3.6 |  |  |  |  |
|  |  |  |  |  |  |  |  |  |  |
| Subcortical | Thalamus | -10 | -16 | 4 | 3.7 | 12 | -14 | 6 | 4.6 |
|  | Lateral globus pallidus | -22 | -4 | -4 | 5.5 | 22 | -6 | 8 | 7.2 |
|  | Putamen | -30 | -16 | 4 | 4.4 |  |  |  |  |
|  |  |  |  |  |  |  |  |  |  |
| Cerebellum | Declive (VI) | -26 | -58 | -22 | 5.8 | 26 | -62 | -24 | 5.2 |
|  | Culmen (V) | -14 | -60 | -18 | 5.0 |  |  |  |  |
|  | Vermis (VIII/pyramis) | 0 | -64 | -32 | 4.2 |  |  |  |  |
|  | Vermis (VI/declive) | 0 | -58 | -24 | 3.6 |  |  |  |  |
