# Supplementary material for: Vocal Accuracy and Neural Plasticity Following Micromelody-Discrimination Training
Source: PLoS One. 2010 Jun 17;5(6):e11181. doi: 10.1371/journal.pone.0011181 (PMC2887372; doi:10.1371/journal.pone.0011181)
Supplement: Table S2 — Connectivity maps associated with right mid-dorsal insula and left ACC BA 24 during simple singing. All peak/cluster ps≤0.05, corrected. ACC = anterior cingulate cortex; BA = Brodmann area; dPMC = dorsal premotor cortex; IPL = inferior parietal lobule; L = left; M1 = primary motor cortex; mid-PMC = mid-premotor cortex; PAC = primary auditory cortex; post = posterior; pre-SMA = pre-supplementary motor area; pSTG = posterior superior temporal gyrus; pSTS = posterior superior temporal sulcus; R = right; RCZa = anterior portion of rostral cingulate zone; SMA = supplementary motor area; STG = superior temporal gyrus; STS = superior temporal sulcus; vPMC = ventral premotor cortex. (0.09 MB DOC) [file pone.0011181.s002.doc]

Table S2. Connectivity maps associated with right mid-dorsal insula and left ACC BA 24 during simple singing.

|  |  | **FUNCTIONAL CONNECTIVITY** | | | | | | | |
| --- | --- | --- | --- | --- | --- | --- | --- | --- | --- |
|  |  | **R mid-dorsal insula (40, 4, 6)** | | | | **L ACC BA 24 (-2, 4, 44)** | | | |
|  |  | ***x*** | ***y*** | ***z*** | ***t*** | ***x*** | ***y*** | ***z*** | ***t*** |
| Auditory | R STG |  |  |  |  | 56 | -6 | 6 | 4.0 |
|  | R pSTG |  |  |  |  | 60 | -42 | 12 | 4.1 |
|  | R STS | 56 | -8 | -10 | 5.3 |  |  |  |  |
|  | R pSTS | 48 | -34 | 6 | 3.6 |  |  |  |  |
|  | L planum temporale |  |  |  |  | -58 | -42 | 24 | 4.9 |
|  | R planum temporale | 62 | -34 | 22 | 5.7 | 64 | -30 | 14 | 5.1 |
| Motor | L ACC - BA 24 | -2 | -4 | 46 | 4.8 | -6 | 8 | 36 | 4.3 |
|  | R ACC - BA 24 | 2 | -4 | 46 | 5.1 |  |  |  |  |
|  | L ACC - BA 32 (RCZa) | -6 | 12 | 40 | 5.0 |  |  |  |  |
|  | R ACC - BA 32 (RCZa) | 10 | 28 | 30 | 4.9 |  |  |  |  |
|  | R SMA |  |  |  |  | 10 | -4 | 50 | 4.5 |
|  | L pre-SMA | -8 | 12 | 44 | 6.5 |  |  |  |  |
|  | R pre-SMA |  |  |  |  | 10 | 10 | 48 | 5.1 |
|  | R M1 |  |  |  |  | 46 | -4 | 44 | 4.7 |
|  | L dPMC |  |  |  |  | -28 | -8 | 68 | 5.2 |
|  | R dPMC |  |  |  |  | 36 | -8 | 54 | 4.4 |
|  | R mid-PMC | 44 | 2 | 48 | 5.0 | 38 | -6 | 48 | 4.7 |
|  | R vPMC | 58 | 10 | 18 | 4.1 |  |  |  |  |
|  | R subcentral |  |  |  |  | 54 | -4 | 6 | 4.1 |
|  | R Rolandic operculum |  |  |  |  | 60 | -14 | 16 | 4.1 |
| Multimodal | L anterior insula | -32 | 14 | 8 | 4.2 |  |  |  |  |
|  | R anterior insula | 38 | 20 | 4 | 4.6 |  |  |  |  |
|  | L mid-dorsal insula | -34 | 2 | 4 | 6.8 |  |  |  |  |
|  | R mid-dorsal insula | 36 | 4 | 12 | 7.0 |  |  |  |  |
|  | L posterior insula | -32 | -20 | 12 | 7.9 |  |  |  |  |
|  | L post. cingulate - BA 31 |  |  |  |  | -4 | -30 | 40 | 3.4 |
|  | R post. cingulate - BA 31 |  |  |  |  | 8 | -22 | 42 | 5.8 |
| Frontal | R inferior frontal - BA 44 | 48 | 14 | 10 | 5.2 |  |  |  |  |
|  | R inferior frontal - BA 45 | 38 | 30 | 0 | 4.3 |  |  |  |  |
|  | L frontal operculum | -48 | 6 | 6 | 4.1 | -48 | 10 | 6 | 5.1 |
|  | R frontal operculum | 40 | 6 | 10 | 5.8 |  |  |  |  |
|  | R superior frontal - BA 9 | 12 | 20 | 58 | 6.4 |  |  |  |  |
|  | L middle frontal - BA 9 | -24 | 40 | 22 | 3.9 |  |  |  |  |
|  | R middle frontal - BA 8 | 42 | 24 | 26 | 4.8 |  |  |  |  |
|  | R inferior frontal junction | 46 | 6 | 28 | 4.2 |  |  |  |  |
| Parietal | R IPL | 60 | -44 | 20 | 4.2 |  |  |  |  |
|  | L parietal operculum | -36 | -26 | 22 | 4.5 |  |  |  |  |
| Subcortical | L thalamus | -14 | -10 | 12 | 5.1 |  |  |  |  |
|  | R thalamus | 16 | -20 | 6 | 7.6 |  |  |  |  |
|  | L putamen | -32 | -6 | 4 | 7.9 |  |  |  |  |
|  | R putamen | 24 | 4 | 4 | 6.8 |  |  |  |  |
|  | L lateral globus pallidus | -22 | -14 | 2 | 3.9 |  |  |  |  |
|  | L medial globus pallidus | -18 | -10 | 0 | 3.4 |  |  |  |  |
